# A Sneak-Peek into the Physician’s Brain: A Retrospective Machine Learning-Driven Investigation of Decision-Making in TAVR versus SAVR for Young High-Risk Patients with Severe Symptomatic Aortic Stenosis

**DOI:** 10.3390/jpm11111062

**Published:** 2021-10-22

**Authors:** Ena Hasimbegovic, Laszlo Papp, Marko Grahovac, Denis Krajnc, Thomas Poschner, Waseem Hasan, Martin Andreas, Christoph Gross, Andreas Strouhal, Georg Delle-Karth, Martin Grabenwöger, Christopher Adlbrecht, Markus Mach

**Affiliations:** 1 Division of Cardiac Surgery, Department of Surgery, Medical University of Vienna, 1090 Vienna, Austria; ena.hasimbegovic@meduniwien.ac.at (E.H.); thomas.poschner@meduniwien.ac.at (T.P.); martin.andreas@meduniwien.ac.at (M.A.); christoph.gross@meduniwien.ac.at (C.G.); 2 Division of Cardiology, Department of Internal Medicine II, Medical University of Vienna, 1090 Vienna, Austria; 3 Center for Medical Physics and Biomedical Engineering, Medical University of Vienna, 1090 Vienna, Austria; laszlo.papp@meduniwien.ac.at (L.P.); denis.krajnc@meduniwien.ac.at (D.K.); 4 Division of Nuclear Medicine, Medical University of Vienna, 1090 Vienna, Austria; marko.grahovac@meduniwien.ac.at; 5 Faculty of Medicine, Imperial College London, London SW7 2AZ, UK; waseem.hasan15@imperial.ac.uk; 6 Vienna North Hospital—Floridsdorf Clinic and the Karl Landsteiner Institute for Cardiovascular and Critical Care Research, 1090 Vienna, Austria; 7 Department of Cardiovascular Surgery, Hospital Hietzing and the Karl Landsteiner Institute for Cardiovascular and Critical Care Research, 1090 Vienna, Austria; andreas.strouhal@gesundheitsverbund.at (A.S.); georg.delle-karth@gesundheitsverbund.at (G.D.-K.); c.adlbrecht@imed19.at (C.A.); 8 Faculty of Medicine, Sigmund Freud University, 1090 Vienna, Austria; martin.grabenwoeger@meduniwien.ac.at; 9 Imed19—Internal Medicine Doebling, 1090 Vienna, Austria

**Keywords:** TAVR, TAVI, iSAVR, aortic stenosis, machine learning, heart team

## Abstract

Transcatheter aortic valve replacement (TAVR) has rapidly become a viable alternative to the conventional isolated surgical aortic valve replacement (iSAVR) for treating severe symptomatic aortic stenosis. However, data on younger patients is scarce and a gap exists between data-based recommendations and the clinical use of TAVR. In our study, we utilized a machine learning (ML) driven approach to model the complex decision-making process of Heart Teams when treating young patients with severe symptomatic aortic stenosis with either TAVR or iSAVR and to identify the relevant considerations. Out of the considered factors, the variables most prominently featured in our ML model were congestive heart failure, established risk assessment scores, previous cardiac surgeries, a reduced left ventricular ejection fraction and peripheral vascular disease. Our study demonstrates a viable application of ML-based approaches for studying and understanding complex clinical decision-making processes.

## 1. Introduction

The development of transcatheter aortic valve replacement (TAVR) over the last two decades, and particularly its use in younger patients, have offered us a glance into the complex interplay between the available study data and the gradual implementation of the new technique into day-to-day practice. Over the last two decades, TAVR was often performed in groups of patients that had not yet been definitively encompassed by the clinical guidelines of the time, and few posed as significant a challenge as young patients. This inspired us to implement a novel approach to examining the factors that guided the clinical decision-making process of heart teams for young patients with severe symptomatic aortic stenosis (AS) during TAVR’s nascent stages.

In less than two decades, TAVR progressed from its inception in the first human patient in 2002 to becoming a well-established widely performed alternative to the isolated surgical aortic valve replacement (iSAVR) [1,2,3]. The first set of ECS valvular disease guidelines published in 2007 merely mentioned TAVR as a promising new technique, without issuing a single TAVR-related recommendation [4]. However, the following year the EACTS and the ESC published a consensus statement listing TAVR as a feasible option for high-risk and inoperable patients with severe symptomatic AS [5]. This recommendation was confirmed by the 2012 ESC valvular heart disease guidelines [6]. These guidelines formalized the requirement for the formation of interdisciplinary heart teams in institutions performing TAVR [6]. The 2014 AHA/ACC guidelines largely echoed the recommendations made by the 2012 ESC guidelines with regards to TAVR-suitable patient collectives [7]. In 2017, the ESC guidelines expanded the TAVR patient collective to intermediate risk patients and some low-risk patients who fulfill certain criteria that favor TAVR [8]. The 2020 ACC/AHA guidelines represent the most extensive set of recommendations published thus far and mark a paradigm shift from a pure risk-related assessment to age- and durability-related considerations [9]. This reflects cumulative improvements in the transcatheter approach, reductions in intervention-related complications and studies on progressively lower risk patient groups. Whereas mechanical devices are still favored for patients younger than 50 years of age, and bioprosthetic valves are the treatment of choice for patients over 65 years of age, all other patients are candidates for both approaches [9]. TAVR has also surpassed iSAVR as the treatment of choice for high-risk patients of any age with a high or prohibitive surgical risk [9].

However, despite the detailed recommendations, there are still significant gaps in evidence, especially pertaining to younger patients. Most studies comparing TAVR to iSAVR have been conducted on older, frail patients, and it is difficult to make inferences regarding survival, adverse events, and the choice of intervention in younger patients, and younger high-risk patients in particular [10,11].

With the advent of machine learning (ML), new tools that expand past traditional statistical models have been made available to make sense of large sets of data and to predict clinical outcomes [12,13]. Once the learning process is complete, ML models should be able to accurately predict outcomes, provided the model is sufficiently accurate and the volume of data sufficient for reaching a given conclusion [14,15]. The surge of supervised machine learning approaches, fueled in part by the increased availability of large sets of data and increased computing power, have revealed a range of potential applications, ranging from disease diagnosis to the identification of potential therapeutic targets and drug development [15,16]. Iterations of ML that could improve or aid clinical decision-making have been examined in areas ranging from infectious disease to emergency care, neurosurgery and even the recent COVID-19 pandemic [17,18,19,20].

The aim of our study was to investigate the decision-making process within the Heart Team in the years where TAVR, a comparatively new technique was progressively developing into a viable alternative to the conventional surgical approach, but no elaborate decision support in the form of guidelines or other scientific analyses existed for young patients. The ML-based analysis was designed to identify features which contribute in great measure to the building of a high-performing ML model predicting the allocation to either TAVR or iSAVR for patients with severe symptomatic AS younger than 75 years of age, as well as to explore how the identified features align with past and current clinical consensus, in order to gain a better understanding of clinical decision-making processes in emerging therapeutic areas. We postulated that these features could be identified with a high accuracy even for a highly imbalanced cohort, such as the one used in our study.

## 2. Materials and Methods

### 2.1. The TAVR Cohort

This study was approved by the regional Ethics Committee of the City of Vienna (ethics approval code: EK18-028-VK). Initially, a total of 532 patients were enrolled in the VIenna CardioThOracic Aortic Valve RegistrY (VICTORY) Registry. The data collection for the VICTORY registry took place between June 2009 and December 2016. The VICTORY registry data was collected in a prospective manner from patients who were set to undergo TAVR at the Hietzing Heart Centre (1140 Vienna, Austria).

After applying the upper age limit of 75 years of age, 105 patients of 75 years of age or less were selected. A further 17 patients had to be excluded from the analysis due to absolute iSAVR contraindications (8 patients exhibited a prohibitive surgical risk, 9 patients were diagnosed with a porcelain aorta). This resulted in 88 patients who represent the TAVR treatment cohort. Prior to the intervention, each patient was evaluated by the institutional Heart Team. Whenever possible or reasonably clear, the Heart team specified the factors in favor of TAVR. Patient preference, although naturally considered, was not reported. The interventions were performed according to the institutional standards in the presence of a cardiologist and a cardiac surgeon. The TAVR valves used across the data collection period included devices from different valve generations developed by Edwards Lifesciences (Edwards Lifesciences, Irvine, CA, USA), Medtronic (Medtronic, Minneapolis, MN, USA), JenaValve (JenaValve Technology GmbH, Munich, Germany) and Symetis (Symetis SA, a Boston Scientific Company, Ecublens, Switzerland).

### 2.2. The iSAVR Cohort

The iSAVR cohort was chosen from a collective of 732 patients younger than 75 years of age who had undergone an isolated surgical valve replacement at the Department of Cardiovascular Surgery of the Hietzing Heart Centre in Vienna, Austria between January 2005 and December 2016. A total of 54 patients had to beexcluded from the analysis due to active endocarditis at the time of surgery. The patient data was collected retrospectively from the institutional quality assurance registry. The data was then processed so that it would correspond to the format of the data in the VICTORY registry. This allowedcomparisons between the cohorts. A total of 74 patients were excluded due to incomplete datasets. Thus, the final size of the somparisiion cohort was reduced to 604 patients. The surgical replacement of the aortic valve was completed according to institutional guidelines, without modifications to the procedure, either via a full or a mini sternotomy (as chosen at the discretion of the operating surgeon). The procedure involved aortic cross-clamping, cardioplegic arrest and was conducted under moderate hypothermic cardiopulmonary bypass (32 °C).

### 2.3. Baseline Parameters

The baseline parameters depicted in Table 1 were collected for all included patients from both cohorts.

### 2.4. Statistical Analysis and the Machine Learning Model

All statistical analyses were performed using the IBM SPSS 26.0 statistic software (SPSS Inc., Armonk, NY, USA). Depending on their distribution, continuous variables were expressed either as a median and interquartile range (IQR) or as mean and standard deviation (±SD). Categorical variables were expressed as absolute numbers and percentages. The means of normally distributed data were compared using the Student’s *t*-test, whereas the Mann-Whitney U test was used for not normally distributed data. Categorical data comparisons were conducted with Pearson’s Chi-Square test or Fisher’s exact test.

In order to build TAVR vs. iSAVR predictive models and to identify relevant features for such predictors, automated machine learning (AutoML ver. 1.0, Dedicaid GmbH, 1090 Vienna, Austria) was utilized. The AutoML builder performed a 100-fold Monte Carlo cross-validation with an 80–20% train-validation split ratio. In each fold, automated data preprocessing including feature redundancy reduction combined with feature ranking and selection as well as class imbalance reduction have been performed prior to machine learning [21,22]. This step was followed by building a mixed, stacked ensemble of multiple prediction models to establish the final prediction model per fold. See Supplemental for details of the AutoML process.

Model performance was estimated by evaluating the validation cases of each fold after model building. Confusion matrix analytics were utilized to calculate true positive (TP), true negative (TN), false positive (FP) and false negative (FN) values in each fold [21]. Lastly, validation sensitivity, specificity, negative and positive predictive values as well as accuracy were calculated from TP, TN, FP and FN values to estimate the overall performance of the machine learning prediction models.

The identification of prominent features as provided by the AutoML approach was performed by calculating how many times each feature in the dataset was selected for machine learning across the 100 cross-validation folds [23]. Feature ranks were normalized to the sum of 1.0 and features with ranks higher than the half-maximum of all ranks were considered as prominent for the classification of TAVR vs. iSAVR label.

## 3. Results

### 3.1. Baseline Characteristics

A total of 692 patients ≤ 75 years of age were included in the study. Of those, 604 (87.3%) received iSAVR and 88 (12.7%) received TAVR. The mean age of the overall cohort was 64.1 years (±9.5 years). There was no significant difference between the age of the TAVR and iSAVR cohort, although the TAVR patients tended to be older than iSAVR patients (iSAVR 63.4 ± 9.8 years vs. TAVR 68.9 ± 5.2 years, *p* = 0.385). At baseline, iSAVR patients had a significantly higher prevalence of dyslipidemia (iSAVR 62.9% vs. TAVR 60.2%, *p* = 0.026) and a significantly lower prevalence of diabetes (iSAVR 27.2% vs. TAVR 40.9%, *p* = 0.012). There were no significant differences in the prevalence of other relevant comorbidities or preconditions, including previous cardiac surgery, atrial fibrillation and the NYHA class. With regards to the operative risk profile, the only significant difference was observed in the Logistic EuroSCORE, which was significantly higher in the iSAVR cohort (iSAVR 21.6% vs. TAVR 17.4%, *p* = 0.032). In addition, these patients also had a significantly higher CHADS-VASC Score (iSAVR 5.8% vs. TAVR 5.2%, *p* = 0.014). A comprehensive overview of the baseline characteristics of our study cohort is shown in Table 2.

### 3.2. Heart Team Decision—Stated Reasons for TAVR

The two most frequent individual reasons for choosing TAVR over iSAVR were the that the surgery would represent a high-risk reoperation in 42 patients (47.7%) and significant respiratory impairment was present in 41 patients (46.6%). Further factors in favor of TAVR that were present in more than a quarter of the patients, in the order of their relative frequency were a severely reduced LVEF (38.6%), severe renal insufficiency (36.4%) and any type ofsubstance abuse (26.1%). In the large majority of cases, i.e., in 74 cases (84.1%), 2 or more reasons were stated in favor of TAVR. A complete list of the leading stated reasons for the choice of TAVR versus iSAVR is shown in Table 3.

### 3.3. Machine Learning Model

**Figure 1:**
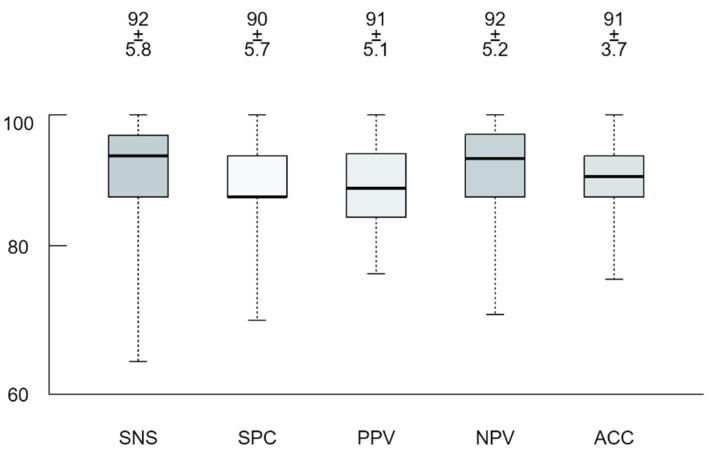
Box-plot Monte Carlo (MC) cross-validation performance of the established model scheme throughout the performance of the top-layer prediction model. Performance values were determined by confusion matrix analytics across all MC folds. SNS—Sensitivity; SPC—Specificity; PPV—Positive Predictive Value; NPV—Negative Predictive Value; ACC—Accuracy; Performance values.

**Figure 2:**
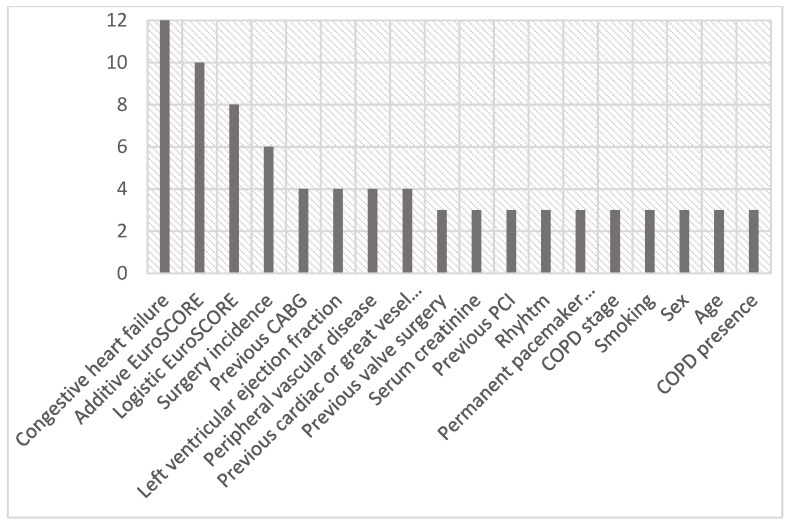
Selected features and their ranks in % as calculated across the MC folds. Ranks represent the relative importance of selected features for model building.

Monte Carlo cross-validation revealed a 0.91 area under the receiver operator characteristics curve with 90% accuracy, 92% sensitivity and 90% specificity to predict TAVR vs. iSAVR within the dataset. See Figure 1 for the box plots of all confusion matrix cross-validation performance values. Overall, eight features were identified as prominent to differentiate TAVR vs. iSAVR (according to the top five relative weight values: 12%, 10%, 8%, 6%, 4%): Congestive heart failure (12%), the additive EuroSCORE (10%), the logistic EuroSCORE (8%), the surgery incidence (6%), previous CABG (4%), LVEF (4%), peripheral vascular disease (4%) and previous cardiac or great vessel surgery (4%). See Figure 2 for the relative weights of the prominent features.

A full description of the machine learning model, as well as a complete list of the feature ranking and the value distributions for the features can be found in the Supplementary Materials.

## 4. Discussion

This study aimed to identify relevant clinical features that contribute to building of a high-performing model for the prediction of the allocation to TAVR versus iSAVR in a young high-risk cohort stratified in the absence of available scientific data or clinical experience. Our cross-validation performance values support our hypothesis that such features can be identified with a high predictive performance even with a highly imbalanced dataset (87% iSAVR vs. 13% TAVR).

The landmark studies that helped establish TAVR in clinical practice were mostly conducted on older patients [10]. The higher patient age in these studies is a natural consequence of a risk-related rather than age-focused approach to TAVR research that was commonly applied over the last two decades, as TAVR indications expanded from inoperable patients to progressively lower risk patient groups [24]. Although retrospective analyses for patients as young as 65 years of age or younger have found similar survival rates and a favorable adverse outcome profile for TAVR compared to iSAVR, extensive prospective randomized controlled trials on younger patients are scarce [25,26]. As illustrated by the 2020 valvular heart disease guidelines, the focus of research is shifting to accommodate age-related considerations [9]. Before TAVR can be deemed safe to use in all age groups, concerns including prosthesis durability, the long-term consequences of TAVR-induced conduction abnormalities, the challenges posed by bicuspid valves, the access to the ostia of the coronary arteries and the long-term outcomes of valve-in-valve procedures need to be addressed [10,24,26,27,28]. Thus, despite the increasing availability of data on TAVR in lower risk patient groups, the young high-risk patients included in our study still constitute a relatively underrepresented collective, making the major determinants of the decision-making process as relevant now as they were then.

According to the official data provided by the institutional Heart Team, most decisions to perform a transcatheter aortic valve replacement in lieu of the conventional surgical approach were based on more than one single factor—in most cases a combination of at least two primary factors. Overall, the most frequently used arguments in favor of TAVR were that the surgery would be a high-risk reoperation, a respiratory impairment was present, the patient had a severely reduced LVEF, renal insufficiency, a history of substance abuse or was severely obese, in that order. For most of these items, these statements are reflected by a higher prevalence or expression of the said item in the TAVR group. The presence of a severe respiratory impairment was a notable exception—patients with COPD were underrepresented in the TAVR cohort.

Our machine learning model boasts both a high accuracy and an exceptional positive predictive value. The factors that seemed to push the Heart Team’s decision towards TAVR according to the weighing of the individual clinical factors in the final model partially reflected their frequency within the abovementioned clinical reasoning. However, the ML model clearly demonstrated that the presence of congestive heart failure was the single most important determinant. Other prominent factors, listed in the order of their importance were the Additive and Logistic EuroSCORE, as well as whether the patient had already received a previous aortic valve replacement. A set of five factors deemed equally important by the ML model followed: previous CABG, a reduced LVEF, the presence of peripheral vascular disease and previous cardiac, intrapericardial or great vessel surgery. The distribution of these main parameters across the two cohorts were mostly uniform, with a more prominent presence in the TAVR cohort. The main exception could be found in the abovementioned risk assessment tools, which were, on average, higher in the iSAVR cohort.

The most prominent factor in the final machine learning model was the presence of congestive heart failure. Although not as highly ranked, a reduced LVEF was also assigned one of the highest feature weights in the model. This seems reasonable in terms of the inclusion and relative significance of the surrogate marker of heart failure—the NYHA class, in commonly used operability assessment tools [29,30,31]. The EuroSCORE II attributes one of the top 5 highest relative significance weights to a highly reduced left ventricular ejection fraction, and a high functional NYHA class is also among the top half of the considered parameters. The STS score similarly includes both parameters, whereas the Logistic EuroSCORE only includes NYHA but not the ejection fraction [32]. In general, a symptomatic left ventricular dysfunction in patients with severe aortic stenosis is present in approximately one fourth of all cases [33]. In the setting of iSAVR, the presence of heart failure is associated with a significantly worse outcome [34]. Similarly, patients with heart failure with a reduced ejection fraction are particularly prone to a worse prognosis following TAVR [35,36,37]. The negative impact of heart failure on the patient outcomes is amplified in the setting of acute decompensated heart failure, with worse survival and overall outcomes for both TAVR and iSAVR [38]. In recent years, the use of TAVR for treating heart failure patients with aortic stenosis has been on the rise, while the mortality following the intervention has experienced a steeper decline compared to iSAVR [39].

A variety of surgical risk assessment scores have been developed to predict the operative mortality of patients undergoing cardiac surgery, including the EUROSCORE—now largely replaced by EuroSCORE II, the Logistic EuroSCORE and the STS Score. It has been found that the Logistic EuroSCORE overestimates the surgical risk for AVR [40]. The STS Score also outperforms the Logistic EuroSCORE in high-risk patients with severe aortic stenosis [41]. Thus, the STS score and EuroSCORE II seem to best predict the mortality of isolated AVR [42]. The two scores used for the surgical risk evaluation in our study that are highly ranked in our model have largely been rendered obsolete by the guidelines, as shown by the clear preference for EuroSCORE II and the STS score [8]. However, EuroSCORE II was only introduced in 2012 and could thus not be retrospectively calculated for the patient cohort in the setting of this study, because it was not considered during the decision process for the entire cohort. The fact that a rigid surgical score seemed to play such an important role in assigning the patients to the treatment groups points to the high reliance of physicians on numerical parameters. However, the score could also act as a surrogate composite marker that indicates the cumulative importance of all the included conditions. In either case, our finding points towards the need for a TAVR/iSAVR specific assessment tool that can be fine-tuned according to clinical findings over time.

An inherent downside of bioprostheses is their degeneration over time, an issue that motivated the development of the TAVR redo technique—valve-in-valve interventions [43]. Our ML model found that whether the valve replacement would replace a native valve with no preceding surgery conducted on the heart, or the patient had undergone a previous valve surgery was a significant factor in choosing TAVR over iSAVR. Newly acquired data seem to support this approach. TAVR has not only proven to be a viable alternative to redo aortic valve surgery, but has also, according to some analyses, surpassed it in terms of early mortality and length of hospital stay [44,45,46,47,48].

A patient collective that comes with a particular set of challenges are those who had already undergone cardiac surgery in the past. This was also a prominent decision-driving factor in our machine learning model. For patients who had undergone previous cardiac surgery, including CABG and previous iSAVR, TAVR seems to perform as well as TAVR in terms of mortality and have a similar adverse outcome distribution as in other patient collectives [25,49,50,51]. Some studies even propose a potential benefit for TAVR in terms of hospitalization time, adverse events, and mortality [44,47,52]. During the data collection period, the 2012 guidelines named previous CABG as one of the few specific factors that could make a patient less suitable for SAVR. This could explain why the heart team physicians leaned towards TAVR in patients with previous cardiac surgery. The 2017 guidelines went on to favor TAVR over SAVR for high-risk elderly patients if a transfemoral access point is feasible, and expanded the TAVR indications to the intermediate risk collective [8]. These guidelines renewed the recommendation for TAVR over SAVR in patients with previous CABG and expanded it to all patients with previous cardiac surgery [8]. The consideration of previous cardiac surgery in our model is therefore in line with this iteration of the guidelines.

Finally, the presence of peripheral vascular disease seemed to speak in favor of TAVR. This is an interesting finding, particularly because vascular complications were one of the two main limitations of TAVR in its early stages. In all guidelines since 2012, the feasibility of a good peripheral vascular access point was listed as one of the determining prerequisites for a successful TAVR intervention. However, our cohort consists of a high number of patients treated via a transapical access with TAVR. In this context, because a vascular access point is not related to the difficulty of the procedure, its role as a surrogate marker of overall poor vessel health and a reduced general condition could have stood at the forefront of the decision-making process.

Surprisingly, previous pacemaker implantation was not a highly significant factor in choosing the appropriate intervention in our ML model. The need for permanent pacemaker implantation, although different between devices, has been one of the most commonly observed adverse events in TAVR patients when compared to their surgical counterparts [52,53,54,55].

The novelty of the approach used in this study compared to conventional clinical statistical analysis is that the output is focused on a different aspect of the collected data. A conventional retrospective study most often focuses on finding the significance in the difference between the distribution of data between multiple groups, and these factors are, in turn, most often chosen based on their established or perceived clinical significance. The ML-driven mode of analysis, however, as applied in this approach, provides us with the relative weight of the chosen feature for the final constructed model.

In general terms, when using conventional statistical methods for clinical studies, the process relies on the knowledge and clinical expertise of the physician in the specific area, as well as previously published data and observed or presumed significance of certain parameters to a set of outcomes to preselect a limited number of parameters. Then, conventional statistical methods are used to examine the significance of the correlation between multiple parameters or between a set of parameters and a given clinical outcome, in order to establish causal relationships between these parameters. This approach works well even with comparatively small amounts of data. Machine learning, on the other hand, can examine vast amounts of data and train itself in order to learn from this data and be able to make predictions. The main advantage of machine learning lies in fields where there is either no substantial previous knowledge on the potentially relevant variables and where complex non-linear inter-variable interactions in massive sets of data make it difficult for an individual examiner to identify these complex relationships. However, the findings of ML often have to be interpreted with caution in order to establish the true relevance of the findings in the given field. A potential way to harness the advantages of both approaches is using them in combination with one another, which should lead to significant leaps in diagnostic and predictive capabilities in the medical field in the future [56].

The main limitations of this study arise from its (methodologically required) retrospective character and the disproportionate representation of the included interventions. However, this must be seen in the light of our study being a retrospective analysis of a historical cohort, as well as the patient preference and clinical center expertise not being accounted for. Considering our findings, which most prominently include congestive heart failure and a reduced LVEF, it might have been pertinent to include low-flow low-gradient and paradoxical low-flow severe AS as individual parameters in the ML model.

## 5. Conclusions

Our final high-performing ML model that retrospectively modeled the allocation to TAVR or iSAVR by the Heart Team physicians at a time when no scientific data or clinical experience has not been available, identified a handful of relevant features, most prominently congestive heart disease. The relevance of the identified factors for building a high-performing ML model partially concurred with and partially deviated from the recommendations of past and current clinical guidelines. The exceptional performance parameters of our machine learning model support our hypothesis that machine learning approaches can help identify factors of particular importance in real-life decision-making processes in difficult clinical scenarios.

## Figures and Tables

**Table 1:** Acquired baseline parameters.

Screening Parameter	Parameter Description
Age	Age in years
Sex	Male/female
Weight	Weight in kilograms
Height	Height in metres
Body Mass Index	
Body Surface Area	
Additive EuroSCORE	
Logistic EuroSCORE	
STS Score	Society of Thoracic Surgeons Score
HAS-BLED-Score	Hypertension, Abnormal Kidney and Liver Function, Stroke, Bleeding History or Predisposition, Labile INR, Elderly, Drugs or Alcohol Concomitantly Score
CHADS-VASC-Score	Congestive Heart Failure, Hypertension, Age, Diabetes mellitus, Stroke, Vascular disease, Age, Sex Category Score
Arterial hypertension	
Smoking	Any period of smoking in lifetime
Active smoker	At the time of intervention
COPD	Presence of COPD according to the STS classification
COPD severity	No symptoms, mild, moderate, severe symptoms
Dialysis	Active dialysis
Creatinine in serum	Creatinine in serum in mg/dL
Diabetes mellitus	No diabetes mellitus Insulin dependent diabetes mellitus Diabetes mellitus treated with dietary measures Diabetes mellitus treated with oral medication Diabetes mellitus diagnosed but not currently treated
Immunosuppression	Active immunosuppression
Peripheral vessel disease	
Cerebrovascular disease	
Previous cerebrovascular event	
Surgery incidence	First valve intervention or redo
Previous CABG	
Previous valve surgery	Previous surgery on any cardiac valve
Previous cardiac surgery—other	
Previous PCI	Previous PCI with or without stent implantation
Myocardial infarction	Previous myocardial infarction
Congestive heart failure	
NYHA class	
LVEF	LVEF in percent
Rhythm	Physiological sinus rhythm Atrial fibrillation Atrial flutter Active pacing
Permanent pacemaker	Previous permanent pacemaker implantation
Mean pressure gradient	In mmHg

Abbreviations: COPD—chronic obstructive pulmonary disease, CABG—coronary artery bypass grafting, PCI—percutaneous coronary intervention, LVEF—left ventricular ejection fraction; STS—Society of Thoracic Surgeons Risk Score; NYHA—New York Heart Association.

**Table 2:** Baseline clinical characteristics of the TAVR and iSAVR cohort.

		Overall *n* = 692	iSAVR < 75 Yrs *n* = 604	TAVR < 75 Yrs *n* = 88	*p* Value
Demographics					
	Age, mean (±SD)	64.1 (9.5)	63.4 (9.8)	68.9 (5.2)	0.385
	Female, *n* (%)	287 (41.5)	237 (39.2)	50 (56.8)	0.370
	Body mass index kg/m^2^, median (IQR)	28.7 (5.5)	28.6 (5.4)	29.2 (6.5)	0.369
Risk profile					
	EuroSCORE, median (IQR)	2.7 (3.7)	1.7 (2.2)	5.9 (5.3)	0.103
	Logistic EuroSCORE, median (IQR)	17.8 (20.4)	21.6 (28.9)	17.4 (19.6)	0.032
	STS score, median (IQR)	4.5 (3.3)	5.8 (4.7)	4.6 (3.2)	0.288
	Incremental risk score, median (IQR)	3 (8)	3 (9)	5 (11.5)	0.889
	HAS-BLED score, median (IQR)	1 (1)	1 (1)	1 (1)	0.085
	CHADS-VASC Score, mean (±SD)	5.3 (1.4)	5.8 (1.4)	5.2 (1.4)	0.014
Chronic health conditions and risk factors					
	Hypertension, *n* (%)	538 (77.7)	464 (76.8)	74 (84.1)	0.160
	Dyslipidemia, *n* (%)	433 (62.6)	380 (62.9)	53 (60.2)	0.026
	Diabetes mellitus, *n* (%)	200 (28.9)	164 (27.2)	36 (40.9)	0.012
	Active smoker, *n* (%)	126 (18.2)	106 (17.5)	20 (22.7)	0.209
	Serum creatinine mg/dL, mean (±SD)	1.1 (0.6)	1.0 (0.4)	1.5 (1.2)	0.433
	Preoperative dialysis, *n* (%)	6 (0.9)	2 (0.3)	4 (4.5)	0.478
	COPD, *n* (%)	217 (31.4)	168 (27.8)	49 (7.1)	0.908
	Peripheral vascular disease, *n* (%)	67 (9.7)	43 (7.1)	24 (27.3)	0.891
	Cerebrovascular disease, *n* (%)	111 (16.0)	87 (14.4)	24 (3.5)	0.478
	Previous cerebrovascular event, *n* (%)	17 (2.5)	7 (1.2)	10 (11.4)	0.143
	Atrial fibrillation, n (%)	119 (17.2)	101 (16.7)	18 (20.5)	0.841
	Previous myocardial infarction, *n* (%)	54 (7.8)	37 (6.1)	17 (19.3)	0.298
	NYHA class III/IV, *n* (%)	367 (53.1)	288 (47.7)	79 (90)	0.820
	Previous PCI, *n* (%)	43 (6.2)	26 (4.3)	17 (19.3)	0.233
	Previous pacemaker implantation, *n* (%)	32 (4.6)	17 (2.8)	15 (17)	0.558
	Previous cardiac surgery, *n* (%)	64 (9.2)	26 (4.3)	38 (43.2)	0.256
	Previous CABG, *n* (%)	34 (4.9)	11 (1.8)	23 (26.1)	0.569
	Previous valve surgery, *n* (%)	34 (4.9)	16 (2.6)	18 (20.5)	
	Previous other cardiac surgery, *n* (%)	17 (2.5)	2 (0.3)	15 (17)	0.161
Preoperative echocardiographic data					
	Mean pressure gradient, mean (±SD)	48 (17.3)	48.6 (17.6)	46.3 (18.3)	0.565
	LVEF in %, mean (±IQR)	52.7 (9.9)	53.4 (9.2)	46.5 (11.9)	0.368

Abbreviations: SD—standard deviation, IQR—interquartile range, Abbreviations: COPD—chronic obstructive pulmonary disease, CABG—coronary artery bypass grafting, PCI—percutaneous coronary intervention, LVEF—left ventricular ejection fraction; STS—Society of Thoracic Surgeons Risk Score; NYHA—New York Heart Association.

**Table 3:** Determining factors forthe choice ofTAVR over iSAVR, as documented by the Heart Team.

	TAVR < 75 Years (*n* = 88)
High-risk reoperation, *n* (%)	42 (47.7)
Significant respiratory impairment, *n* (%)	41 (46.6)
Severely reduced LVEF, *n* (%)	34 (38.6)
Severe renal insufficiency, *n* (%)	32 (36.4)
Substance abuse, *n* (%)	23 (26.1)
Adipositas per magna, *n* (%)	16 (18.2)
Valve-in-Valve procedure, *n* (%)	13 (12.5)
Neurological impairment, *n* (%)	12 (14.8)
Hepatopathy, *n* (%)	10 (11.4)
History of radiation to the chest, *n* (%)	9 (10.2)
Severe mental disorder, *n* (%)	9 (10.2)
Pulmonary hypertension, *n* (%)	7 (8.0)
Frailty, *n* (%)	3 (3.4)
Severe rhythm disorder, *n* (%)	2 (2.3)
History of severe bleeding, *n* (%)	1 (1.1)
Other, *n* (%)	17 (19.3)
Patients with 2 or more reasons listed above	74 (84.1)
Patients with 3 or more reasons listed above	35 (39.8)

Abbreviations: LVEF—left ventricular ejection fraction.

## Data Availability

The data presented in this study are available on reasonable request from the corresponding author.

## Supplementary Materials

The following are available online at https://www.mdpi.com/article/10.3390/jpm11111062/s1: supplementary file describing the machine learning model, as well as the data processing steps.
