# Supplementary material for: A Sneak-Peek into the Physician’s Brain: A Retrospective Machine Learning-Driven Investigation of Decision-Making in TAVR versus SAVR for Young High-Risk Patients with Severe Symptomatic Aortic Stenosis
Source: J Pers Med. 2021 Oct 22;11(11):1062. doi: 10.3390/jpm11111062 (PMC8622882; doi:10.3390/jpm11111062)
Supplement: Supplementary file 1 [file jpm-11-01062-s001.zip › jpm-1399607-supplementary.pdf]

SUPPLEMENTARY MATERIAL

AUTOMATED MACHINE LEARNING ANALYSIS

Tabular data submission for Dedicaid AutoML services (Medical University of Vienna on-site, offline variant) was performed by Dedicaid user markus.mach@meduniwien.ac.at on 3/23/2021, 9:54:03 AM to build and cross-validate automated data preprocessing and mixed, stacked ensemble learning pipelines for predicting reference label "Procedure (0-iSAVR, 1-TAVR)". For the basic properties of the analysis see Table S1.

Table S1. Properties of the automated machine learning (AutoML) analysis of this study.

Data name	iSAVR-vs-TAVR.xlsx
Data size	692 samples, 28 features
Date of analysis	3/23/2021, 9:54:03 AM
Duration of analysis	4h 33m 45s
Submitted by	markus.mach@meduniwien.ac.at
Dedicaid AutoML version	1.0 (MUW on-site, offline variant)

METHODS

Data

The input dataset was composed of 692 samples and 28 features. The selected reference label for the cross-validation was "Procedure (0-iSAVR, 1-TAVR)" having subgroups of 0 (87.2%) and 1 (12.7%) label outcomes.

Cross-Validation

Monte Carlo (MC) cross-validation scheme was applied with 80% training and 20% validation ratios across 100 folds [1]. Each fold had unique training-validation configurations. MC split resulted in 554 samples per fold in the training set. The validation set of each fold contained 69 samples per reference label (138 overall). The validation samples were equally subsampled to ensure that none of the label outcomes are over or underrepresented during the cross-validation.

Preprocessing

The data underwent preprocessing steps in each fold before performing machine learning (ML) analysis. Preprocessing resulted in average 23 features across all MC folds. For the preprocessing steps and their parameters, see Table S2.

Table S2. Preprocessing step algorithms as well as their parameter values performed in all Monte Carlo folds before machine learning. FN - Feature Normalization; KE - Kernel-based Feature Engineering; SRR - Smart Redundancy Reduction; SSYN - Sample Synthetizer.

Preprocessing step	Algorithm	Parameter	Value	Reference
1	FN	Normalization type	Mean-Deviation	[2]
2	KE	Kernels applied	Gaussian; Polynomial; Tanh	[3]
3	FN	Normalization type	Mean-Deviation	[2]
4	SRR	Redundancy Threshold (Covariance)	0.85	[4]
5	SSYN	Oversampling ratio (majority subgroup)	1.017	[5]
		Sampling technique	SMOTE	

Note: In case a Kernel Engineering (KE) step is involved in the preprocessing, an additional Feature Normalization (FM) is performed to ensure that both original and engineered features are within comparable ranges.

Machine Learning Layer 1

Various machine learning algorithms were established in each fold to minimize the effect of algorithm bias (4. Each model was trained by randomly selecting 80% of the preprocessed training data per MC fold. For details of the ML algorithms, see Table S3.

Table S3. Machine learning (ML) algorithms in the first ML layer with their parameters and value ranges across Monte Carlo (MC) folds. Occurrence of each ML type is represented in percentages across MC folds. BYS – Bayesian Classifier; MGWC – Multi-Gaussian Weighted Classifier; RF – Random Forest Classifier; SVM – Support Vector Machine Classifier;

ML Algorithm	Parameter	Value Range	Occurrence	Reference
BYS	–	–	21.06%	[6]
MGWC	Initial value multiplier	1 – 10	25.51%	[7]
	Maximum iterations	23000 – 115000		
	Negative weights allowed	false, true		
	Scale value multiplier	0.1 – 50		
	Tolerance	0.00001 – 0.0001		
RF	Bag fraction	0.8 – 0.99	27.59%	[4]
	Bagging method	equalized, normal		
	Boosting	none, adaboost		
	Maximum tree depth	5 – 14		
	Minimum samples in leaves	3 – 7		
	Node feature selection method	none		
	Number of random features per node	5		

	Number of selected trees	101 – 201		
	Number of trees to build	301 – 1001		
	Tree quality metric	gain, gini		
	Tree selection method	0		
SVM	Learning rate	0.001 – 0.01	25.81%	[8]
	Maximum iterations	1000 – 5000		

Machine Learning Layer 2

Meta-training sets were created by evaluating the samples of the preprocessed training set in each MC fold by the trained models in ML layer 1. In order to create the meta-training set, the prediction results of each trained model in ML layer 1 were handled as feature values of the given training sample. The meta-training set was the input for training the second ML layer prediction models. These models were trained to identify patterns in the prediction of the first ML layer models to result in mixed super learners [9]. For the parameters of the second layer ML algorithms see Table S4.

Table S4. Machine learning (ML) algorithms in the second ML layer with their parameters and value ranges across Monte Carlo (MC) folds. Occurrence of each ML type is represented in percentages across MC folds. MGWC – Multi-Gaussian Weighted Classifier; RF – Random Forest Classifier; SVM – Support Vector Machine Classifier;

ML Algorithm	Parameter	Value Range	Occurrence	Reference
MGWC	Initial value multiplier	10	33.33%	[7]
	Maximum iterations	5000 – 16000		
	Negative weights allowed	false, true		
	Scale value multiplier	1 – 5		
	Tolerance	0.0001		
RF	Bag fraction	0.8 – 0.99	33.33%	[4]
	Bagging method	equalized, normal		
	Boosting	none, adaboost		
	Maximum tree depth	5		
	Minimum samples in leaves	3 – 7		
	Node feature selection method	none		
	Number of random features per node	5		
	Number of selected trees	201		
	Number of trees to build	501 – 1001		
	Tree quality metric	gain, gini		
	Tree selection method	none		
SVM	Learning rate	0.001 – 0.01	33.33%	[8]
	Maximum iterations	1000 – 5000		

Top-Layer Model

Combination of the prediction results of the second layer ML models was performed by weighted majority voting to provide the final prediction of the model scheme. Weighting of each ML Layer 2 model was calculated based on training performance. In addition, ML Layer 2 models having less training performance than the median of all ML layer 2 model training performances had weight 0 in the final vote.

RESULTS

Cross-Validation Performance

Model prediction performance was estimated via the MC cross-validation scheme utilizing confusion matrix analytics [10]. True positive, true negative, false positive and false negative confusion matrix entries were calculated by evaluating the validation samples by the established model pipeline in each fold. Sensitivity, specificity, accuracy, positive predictive as well as negative predictive values were calculated across the MC fold validation results. For the average cross-validation performance of ML Layer 1 and 2 models see Table S5 and Table S6 respectively. For the cross-validation results of the final (top-layer) prediction models as well as for the summary of the evaluation, see Table S7 and Figure S1.

Table S5. Average Monte Carlo (MC) cross-validation performance (%) of ML Layer 1 (ML-1) predictive models as determined by confusion matrix analytics across all MC folds. BYS – Bayesian Classifier; MGWC – Multi-Gaussian Weighted Classifier; RF – Random Forest Classifier; SVM – Support Vector Machine Classifier; SNS – Sensitivity; SPC – Specificity; PPV – Positive Predictive Value; NPV – Negative Predictive Value; ACC – Accuracy; OCC – Occurrence. Performance and occurrence values are in percentages.

	SNS	SPC	PPV	NPV	ACC	OCC
**BYS**	76	92	91	80	84	21
**MGWC**	90	84	85	90	87	26
**RF**	91	91	91	92	91	28
**SVM**	81	87	87	82	84	26

Table S6. Average Monte Carlo (MC) cross-validation performance (%) of ML Layer 2 (ML-2) predictive models as determined by confusion matrix analytics across all MC folds. MGWC – Multi-Gaussian Weighted Classifier; RF – Random Forest Classifier; SVM – Support Vector Machine Classifier; SNS – Sensitivity; SPC – Specificity; PPV – Positive Predictive Value; NPV – Negative Predictive Value; ACC – Accuracy; OCC – Occurrence. Performance and occurrence values are in percentages.

	SNS	SPC	PPV	NPV	ACC	OCC
**MGWC**	84	89	89	86	86	33
**RF**	93	90	91	93	92	33
**SVM**	80	90	89	82	85	33

Table S7. Performance Monte Carlo (MC) cross-validation performance of the established model scheme throughout the performance of the top-layer prediction model. Performance values were determined by confusion matrix analytics across all MC folds. MGWC – Multi-Gaussian Weighted Classifier; RF – Random Forest Classifier; SVM – Support Vector Machine Classifier; SNS – Sensitivity; SPC – Specificity; PPV – Positive Predictive Value; NPV – Negative Predictive Value; ACC – Accuracy; OCC – Occurrence. Performance values are in percentages. LQ – Lower quartile; UQ – Upper Quartile; Dev – Deviation.

	Min	LQ	Median	UQ	Max	Mean	Dev
**SNS**	64.7	88.23	94.11	97.05	100	91.82	5.78
**SPC**	70.58	88.23	88.23	94.11	100	90.23	5.72
**ACC**	76.47	88.23	91.17	94.11	100	91.02	3.71
**PPV**	77.27	85.35	89.47	94.44	100	90.78	5.14
**NPV**	71.42	88.23	93.75	97.22	100	92.21	5.15

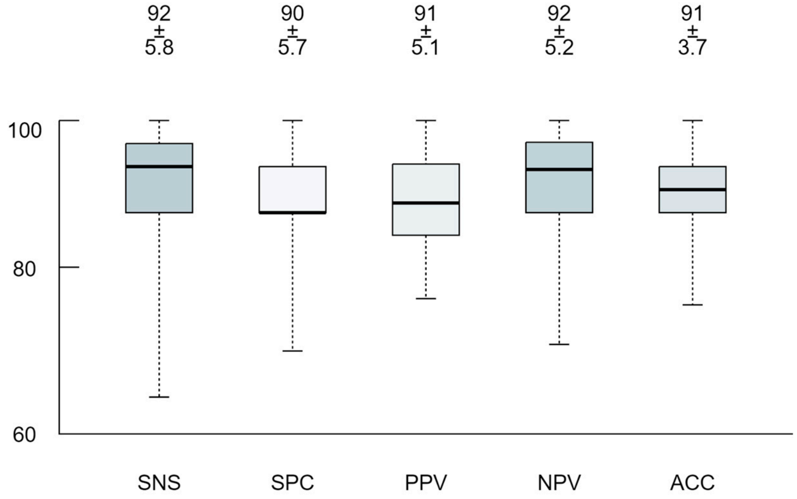

Figure S1. Box-plot Monte Carlo (MC) cross-validation performance of the established model scheme throughout the performance of the top-layer prediction model. Performance values were determined by confusion matrix analytics across all MC folds. SNS – Sensitivity; SPC – Specificity; PPV – Positive Predictive Value; NPV – Negative Predictive Value; ACC – Accuracy; Performance values are in percentages.

Feature Ranking

Feature ranking and selection was performed as part of the data preprocessing steps of each fold (see Sec. Preprocessing). The final feature ranking was calculated as the mean of all feature rankings across the MC folds.

Table S8. Selected features and their ranks as calculated across the MC folds by Smart Redundancy Reduction (SRR - see Table S2) as well as their respective value distributions. Ranks represent the relative importance compared to one another. Features are ordered by ranks. Rank values are in percentages.

Feature Name	Ranking	Histogram
CHF	12%	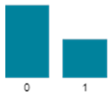

Add ES	10%	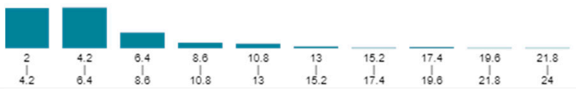
ES	8%	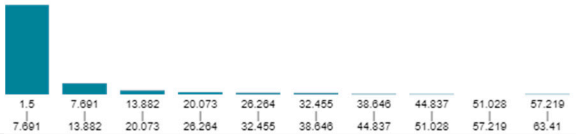
OP Incidence (1-First, 2-Reop)	6%	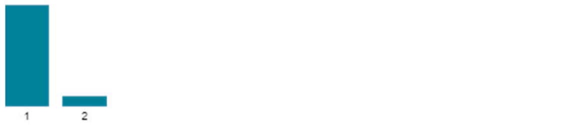
Previous CABG	4%	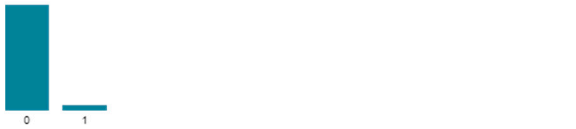
LVEF prc	4%	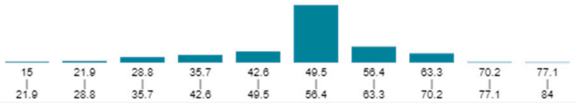
PVD (0-no, 1-yes)	4%	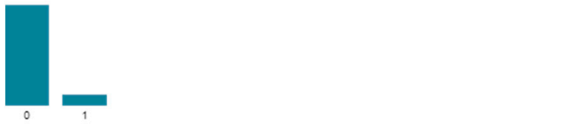
Previous other Cardiac - Intrapericardial or Great Vessel [PrOthCar]	4%	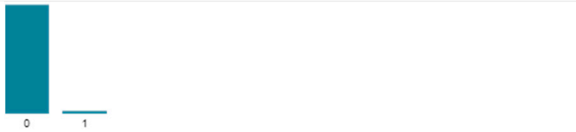
Previous Valve	3%	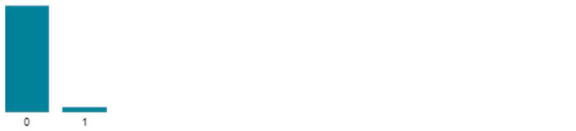
Crea	3%	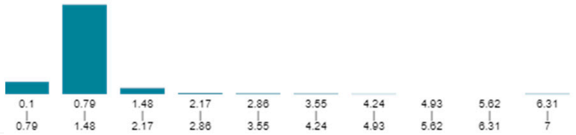
St.p. PCI	3%	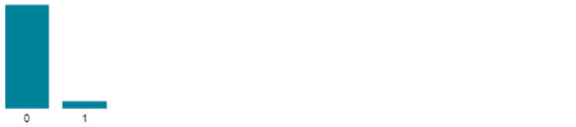
Rhythm(0-Sinus, 1- Afib/-flutter, 2-Paced	3%	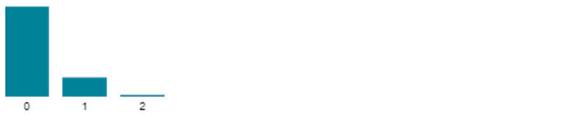
Perm. Pacemaker (0-no, 1 yes)	3%	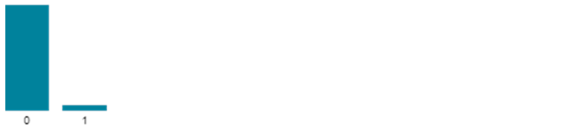

COPD (0-no, 1-mild, 2-moderate, 3-severe)	3%	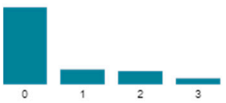
Smoking (0-never, 1-yes)	3%	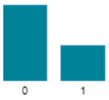
Sex (1-F, 0-M)	3%	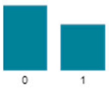
Age (at DOI)	3%	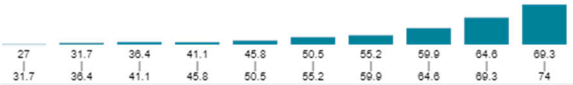
COPD (0-no, 1-yes)	3%	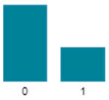
CVD (0-no, 1-yes)	3%	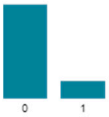
Dialysis (0-no, 1-yes)	2%	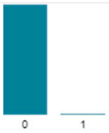
St.p. MCI	2%	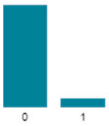
Height (cm)	2%	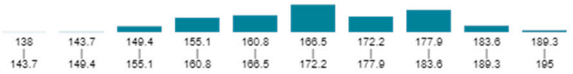
DM (0-no, 1-diet, 2-oral, 3-insulin 4-not treated)	2%	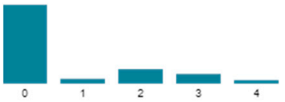
Immunsuppr. Thx (0-no, 1-yes)	2%	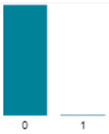
Current Smoker [SmokCurr]	2%	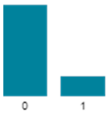

<table>
<tr><td>Weight (kg)</td><td>1%</td><td>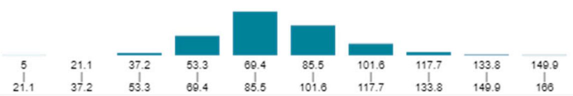
</td></tr>
<tr><td>BMI</td><td>1%</td><td>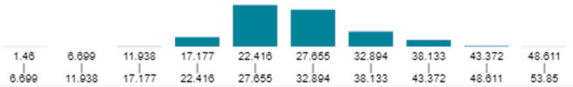
</td></tr>
<tr><td>BSA</td><td>1%</td><td>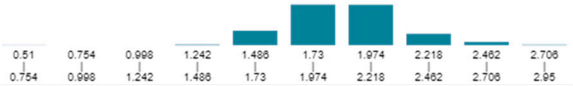
</td></tr>
</table>

References

1. Papp L, Spielvogel CP, Rausch I, Hacker M, Beyer T. Personalizing Medicine Through Hybrid Imaging and Medical Big Data Analysis. Front Phys [Internet]. 2018 Jun 7;6. Available from: https://www.frontiersin.org/article/10.3389/fphy.2018.00051/full
2. Han J, Pei J, Kamber M. Data Mining: Concepts and Techniques [Internet]. Elsevier Science; 2011. (The Morgan Kaufmann Series in Data Management Systems). Available from: https://books.google.at/books?id=pQws07tdpjoC
3. Souza CR. Kernel Functions for Machine Learning Applications [Internet]. 2020. Available from: http://crsouza.blogspot.com/2010/03/kernel-functions-for-machine-learning.html
4. Papp L, Spielvogel CP, Grubmüller B, Grahovac M, Krajnc D, Ecsedi B, et al. Supervised machine learning enables non-invasive lesion characterization in primary prostate cancer with [68Ga]Ga-PSMA-11 PET/MRI. Eur J Nucl Med Mol Imaging [Internet]. 2020 Dec 19; Available from: http://link.springer.com/10.1007/s00259-020-05140-y
5. Amin A, Anwar S, Adnan A, Nawaz M, Howard N, Qadir J, et al. Comparing Oversampling Techniques to Handle the Class Imbalance Problem: A Customer Churn Prediction Case Study. IEEE Access. 2016;4(October):7940–57.
6. Langarizadeh M, Moghbeli F. Applying Naive Bayesian Networks to Disease Prediction: a Systematic Review. Acta Inform Medica [Internet]. 2016;24(5):364. Available from: http://www.scopemed.org/?mno=247785
7. Papp L, Pötsch N, Grahovac M, Schmidbauer V, Woehrer A, Preusser M, et al. Glioma survival prediction with combined analysis of in vivo 11C-MET PET features, ex vivo features, and patient features by supervised machine learning. J Nucl Med. 2018;59(6):892–9.
8. Gao X, Chu C, Li Y, Lu P, Wang W, Liu W, et al. The method and efficacy of support vector machine classifiers based on texture features and multi-resolution histogram from18F-FDG PET-CT images for the evaluation of mediastinal lymph nodes in patients with lung cancer. Eur J Radiol [Internet]. 2015;84(2):312–7. Available from: http://dx.doi.org/10.1016/j.ejrad.2014.11.006
9. van der Laan MJ, Polley EC, Hubbard AE. Super Learner. Stat Appl Genet Mol Biol [Internet]. 2007 Jan 16;6(1). Available from: https://www.degruyter.com/view/j/sagmb.2007.6.issue-1/sagmb.2007.6.1.1309/sagmb.2007.6.1.1309.xml
10. Stehman S V. Selecting and interpreting measures of thematic classification accuracy. Remote Sens Environ [Internet]. 1997 Oct;62(1):77–89. Available from: https://linkinghub.elsevier.com/retrieve/pii/S0034425797000837
